# Immunisation of the somatostatin gene alters hypothalamic-pituitary-liver gene expressions and enhances growth in Dazu black goats

**DOI:** 10.5713/ab.24.0121

**Published:** 2024-06-26

**Authors:** Ge Qin, Shiyong Fang, Xianqing Song, Li Zhang, Jiazhuo Huang, Yongfu Huang, Yanguo Han

**Affiliations:** 1 College of Animal Science and Technology, Chongqing Key Laboratory of Forage & Herbivore, Chongqing Engineering Research Centre for Herbivores Resource Protection and Utilization, Southwest University, Chongqing 400715, China

**Keywords:** Dazu Black Goats, DNA vaccine, Hormones, Somatostatin, Transcriptome Sequencing

## Abstract

**Objective:**

Somatostatin (SS) plays important regulatory roles in animal growth and reproduction by affecting the synthesis and secretion of growth hormone (GH). However, the mechanism by which SS regulates growth and development in goats is still unclear.

**Methods:**

In this study, we randomly selected eight 7-month-old Dazu black goats (DBGs) of similar body weight and equally assigned four bucks as the immunised and negative control groups. The immunised group received the Salmonella typhi attenuated vaccine *X9241* (ptCS/2SS-asd) orally, whilst the negative control group received the empty vector vaccine *X9241* (pVAX-asd) orally.

**Results:**

The SS concentration in the serum of goats in the immunised group was significantly lower than that in the negative control group, and the daily gain was significantly higher (p<0.05). SS-14 DNA vaccine immunisation resulted in significantly higher concentrations of growth-related hormones such as GH-releasing hormone and insulin growth factor 1 (IGF-1) in the serum of goats (p<0.05). RNA-seq analysis of hypothalamus of oral SS-14 DNA vaccine and negative control DBGs identified 31 differentially expressed genes (DEGs). Pituitary gland identified 164 DEGs. A total of 246 DEGs were detected in the liver by RNA-seq. Gene ontology of DEGs was enriched in mitochondrial envelope, extracellular region, receptor binding and cell proliferation. The biological metabolic pathways associated with DEGs were explored by Kyoto encyclopedia of genes and genomes analysis. DEGs were associated with metabolic pathways, oxidative phosphorylation, vitamin digestion and absorption and galactose metabolism. These candidate genes (e.g. *DGKK*, *CYTB*, *DUSP1*, and *LRAT*) may provide references for exploring the molecular mechanisms by which SS promotes growth and development.

**Conclusion:**

Overall, these results demonstrated that the SS DNA vaccine enhanced the growth of DBGs by altering growth-related hormone concentrations and regulating the expression of growth-related genes in the hypothalamic–pituitary–liver axis.

## INTRODUCTION

Immune neutralisation techniques targeting hormones are increasingly being used in studies to improve animal performance. Amongst them, active and passive immunisation against somatostatin (SS), which is a regulatory peptide secreted by the hypothalamus and widely distributed in many tissues, has been shown to improve animal growth [1]. SS includes two types, namely, SS-14 and SS-28. In growth endocrine regulation, SS exerts a negative regulatory effect on the growth axis mainly by inhibiting the spontaneous secretion of growth hormone (GH) *in vivo* and the secretion induced by various stimulating factors [2]. SS operates by binding to SS receptors. Many studies have shown that the antibodies produced by animals treated with SS neutralising immunisation techniques can bind to endogenous SS and block the binding of SS to the receptor. Such treatment also accelerates the clearance of SS from the blood circulation by the action of the liver and promotes the release of various hormones, such as GH and insulin growth factor 1 (IGF-1), which improves the growth rate of animals [3]. Several DNA vaccines encoding SS genes have been developed, and SS-14 gene vaccines immunised in mice, pigs and Hu lambs were effective in reducing peripheral blood SS levels and improving their growth rate and lactation [4]. However, SS-28 vaccination was ineffective in promoting animal growth and lactation [5]. Attenuated cholera is an ideal candidate for the delivery of SS-14 DNA vaccines because it can transfer plasmids into host cells and trigger strong humoral and cellular responses. More importantly, an attenuated cholera-delivered DNA vaccine does not require protein antigen extraction and synthesis, which makes it more convenient and less costly [6]. Our laboratory successfully developed novel recombinant oral SS-14 DNA vaccine C500 (ptCS/2SS-14-asd) and SS-28 DNA vaccine C500 (ptCS/2SS-28-asd) delivered by attenuated Salmonella cholerae into female Sprague Dawley (SD) rats in the previous stage and found that the oral SS-14 DNA vaccine induced strong humoral immune responses [5].

The meat production performance of goats in China lacks superior breeds and currently relies on crossbreeding to improve the meat productivity of goats. Here, we propose the conjecture that SS DNA vaccine immunisation technology can promote goat growth and work on the application of hormone immune neutralisation technology to practical production. Most local goat breeds in China have high litter sizes but slow growth rates, such as Dazu black goats (DBGs), Yimeng black goats and Cangshan black goats. DBGs are a goat breed with pure black wool and stable genetics located in southwest China [7]. DBGs are an excellent breed of goat with skin and meat, which has high reproductive performance, outstanding multilambing, a lambing rate of 218% in first lambing, 272% in lambing and a lamb survival rate of ≥90% [8]. Moreover, the protein content of DBG meat is above 19.6%, and the cholesterol content is 75% lower than that of pork, which is enjoyed by people because of its delicious meat quality [9]. Growth traits are one of the most important economic traits of meat animals, and a large gap exists in production performance compared with the reproductive performance of DBGs. Therefore, the production performance of DBGs needs to be improved. SS secreted by the hypothalamus binds to its receptors and exerts a broad regulatory effect on animal growth and reproduction by modulating pituitary GH secretion [4]. In addition, GH is inhibited by feedback from peripheral tissues, especially liver-derived IGF-1 [10]. And how SS affects growth-related signaling molecules through the hypothalamic-pituitary-liver axis urgently needs to be explored. In our laboratory, we have developed a novel SS-14 DNA vaccine fused to the tPA signal peptide and CpG adjuvant, and delivered by an attenuated cholerae streptococcus strain (C500) without antibiotic resistance genes [4]. This vaccine was successful in increasing serum prolactin (PRL) and GH levels and improving lactation performance in goats [4].

In this study we hypothesized that immunisation of DBGs with SS-14 DNA vaccine would enhance growth rate, assuming that SS-14 DNA vaccine regulates the growth performance of DBGs via hypothalamic-pituitary-growth axis feedback in animals. In this study, we selected bucks with DBGs of similar weight and age for months. They were immunised orally with attenuated Salmonella typhimurium-delivered SS vaccine *X9241* (ptCS/2SS-asd), and controls were immunised orally with empty vector vaccine *X9241* (pVAX-asd). Booster immunisation was administered at the 2nd and 4th weeks after the initial immunisation. The growth rate and changes in serum levels of growth-related hormones were analysed under different treatments, and hypothalamus, pituitary and liver tissues were collected from goats to screen growth-related genes using RNA-seq. Therefore, the present study will contribute to hormonal immunomodulation techniques to improve the performance of livestock and help elucidate the mechanism by which SS promotes the growth performance of DBGs through the hypothalamic–pituitary–liver axis.

## MATERIALS AND METHODS

### Ethics statement

All animal work in this study met the minimum standards of animal welfare as described in the International Guiding Principles for Biomedical Research involving Animals (at https://grants.nih.gov/grants/olaw/Guiding_Principles_2012.pdf). All goats involved in this study followed the regulations of the Southwestern University Institutional Animal Care and Use Committee (IACUC-20210515-05). All efforts were made to alleviate animal suffering and to improve their quality of life.

### Immunisation and sample collection

In this study, eight 7-month-old bucks of similar weight were selected from the Southwestern University farm, and the bucks were equally assigned to four bucks as the immunised and negative control groups. These bucks were reared under similar conditions and fed a commercial full-value pelleted diet purchased from Pizhou Xiaohe Technology Development Ltd. (Xuzhou, China). Nutrient levels for commercial complete granulated feed are shown in Table 1 (all nutrient levels are actual measured values, except for the calculated values for digestibility). We fed the bucks twice a day, at 7 a.m. and 3 p.m. In order to avoid the influence of the feed intake of the experimental animals on the experimental results, we strictly controlled the daily feed intake of the bucks in the negative control group and the immunised group to be consistent. According to the manufacturer’s instructions, each buck was fed 2 kg of full-price pelleted feed per day throughout the experiment. The bucks were able to take all the feed at each feeding. The goats were weighed before the morning feed. Thirty minutes before vaccination, 15 mL of 7.5% sodium bicarbonate solution was administered to each goat to neutralize stomach acid. Immune group bucks were immunised orally with attenuated Salmonella typhimurium-delivered SS vaccine *X9241* (ptCS/2SS-asd) at a dose of 5×10^9^ CFU, whilst the negative control group was immunised orally with empty vector vaccine *X9241*(pVAX-asd). Booster vaccination was administered at the 2nd and 4th weeks after the initial immunisation. After initial or booster immunisation, experimental bucks did not show any signs of inflammation or disease at the injection site. Peripheral blood was collected from the goats before the initial immunisation and at the 4th and 8th weeks after the immunisation and slaughtered at the Southwest University goat farm at the 8th week after the initial immunisation. Three goats were randomly selected from each of the immunised and negative control groups and were bled to death according to the Southwestern University Institutional Animal Care and Use Committee (IACUC-20210515-05). Hypothalamic, pituitary and liver tissues were collected from the experimental and negative control groups of goats and stored in liquid nitrogen.

### Calculation of daily weight gain of Dazu black goats

Before the initial immunisation, the DBGs in the immunised and negative control groups were weighed, and the calculation of daily weight gain (g/d) per buck was performed at the 4th and 8th weeks after the initial immunisation.

### Detection of anti-somatostatin antibody

In this study, specific anti-SS antibodies were tested by an introductory enzyme-linked immunosorbent assay (ELISA) method (Cusabio Biotech, Wuhan, China). Briefly, SS-14 antigen was coated on a 96-well ELISA plate, diluted in bicarbonate buffer (pH 9.6) and incubated overnight at 4°C. PBST was washed three times with 0.05% Tween-20 in phosphate-buffered saline, after which PBST was closed with 1% bovine serum albumin solubilised at 37°C for 1 h. Serum samples from the subject goats were serially diluted in PBST (1:25, 1:50, 1:100, 1:200, 1:400, 1:800, 1:1,600 and 1:3,200) and then, 100 μL was added to each well and incubated at 37°C for 1 h. Negative control serum samples from preimmunised goats were used. Specific SS-binding antibodies were tested by adding horseradish peroxidase-labelled rabbit anti-rat immunoglobulin G (IgG) secondary antibody (Abbkine, Inc., Redlands, CA, USA) diluted in PBST (1:5,000) and subsequently incubated for 1 h at 37°C. Samples were incubated at 37°C for 25 min, and the enzymatic reaction was performed using tettramethylbenzidine substrate. H_2_SO_4_ of 2 M was applied to stop the reaction, and the absorbance at 450 nm was measured by a Bio-Rad iMark microplate absorbance reader (Bio-Rad, Hercules, CA, USA). The endpoint titre of these samples was determined as the reciprocal of the highest serum dilution where the absorbance was greater than the mean plus two standard deviations (SD) of the negative control sample (serum samples from all pre-immunized individuals) at the same dilution. If the absorbance of a serum sample at the dilution at 1:25 was lower than the mean of negative control samples (serum samples from all pre-immunized individuals) plus two standard deviations at the dilution at 1:25 and its absorbance is less than 0.2, the antibody titer of this sample is 0.00 under our existing criteria [13].

### Measurement of serum growth-related hormone levels

Serum concentrations of GH-releasing hormone (GHRH), SS, IGF-1, GH, and ghrelin were measured by radioimmunoassay in bucks (Sino-British Institute of Biotechnology, Beijing, China), and polyclonal rabbit anti-GHRH, SS, IGF-1, GH, and ghrelin antibodies (Sino-UK Institute of Biological Technology, Beijing, China) and donkey anti-rabbit IgG antibodies (Sino-UK Institute of Biological Technology, China) were used. The intra- and interassay coefficients of variation were less than or equal to 15%, respectively.

### RNA extraction, library construction and sequencing

Total RNA was extracted from collected hypothalamic, pituitary and liver tissue samples using the TRIzol kit (Invitrogen, Carlsbad, CA, USA) in accordance with the protocol of the manufacturer. Samples from the hypothalamus of the immune group were named B1-1, B1-2, and B1-3; samples from the pituitary gland were named P1-1, P1-2, and P1-3; and samples from the liver were named L1-1, L1-2, and L1-3. Samples from the hypothalamus of the negative control group were named B2-1, B2-2, and B2-3; samples from the pituitary gland were P2-1, P2-2, and P2-3; and samples from the liver were L2-1, L2-2, and L2-3. RNA quality was assessed on an Agilent 2100 Bioanalyser (Agilent Technologies, Palo Alto, CA, USA) and examined using RNase-free agarose gel electrophoresis. After total RNA was extracted, eukaryotic mRNA was enriched by oligo(dT) beads, whilst prokaryotic mRNA was enriched by removing rRNA with a Ribo-Zero Magnetic Kit (Epicentre, Madison, WI, USA). Then, the enriched mRNA was fragmented into short fragments using fragmentation buffer and reverse transcribed into cDNA with random primers. Second-strand cDNA was synthesised by DNA polymerase I, RNase H, dNTP and buffer. Next, the cDNA fragments were purified with a QiaQuick PCR extraction kit (Qiagen, Venlo, The Netherlands), end repaired, poly(A) added and ligated to Illumina sequencing adapters. The ligation products were size selected by agarose gel electrophoresis, polymerase chain reaction (PCR) amplified and sequenced using Illumina HiSeq2500 by Gene Denovo Biotechnology Co. (Guangzhou, China).

### Transcriptome alignment and functional analysis

Quality control was analysed using FASTP (version 0.18.0) software for raw sequencing reads and filtering of low-quality data [12]. The parameters included that removing reads containing adapters, removing reads containing more than 10% of unknown nucleotides (N) and removing low quality reads containing more than 50% of low quality (Q-value ≤20) bases. Clean reads were aligned to the goat reference genome using Hisat2 (v2.0.4) [13]. The mapped reads of each sample were assembled by using StringTie v1.3.1 [14] in a reference-based approach. For each transcription region, a FPKM (fragment per kilobase of transcript per million mapped reads) value was calculated to quantify its expression abundance and variations, using RSEM software [15]. Gene expression levels were quantified using fragments per kilobase per million mapped reads. DESeq2 (v1.10.1) [16] was then used to determine DEGs between immune and control samples. Differentially expressed mRNAs with a false discovery rate (FDR) <0. 05 and |log2 (fold change)|>1 were considered significant. Gene ontology (GO) (https://www.geneontology.org/) was used to retrieve biological process, molecular function and cellular component terms for obtaining GO annotations of DEGs. Kobas (https://kobas.cbi.pku.edu.cn/home.do) software was used to analyse the Kyoto encyclopedia of genes and genomes (KEGG) pathway with a corrected p value (FDR) cut-off of 0.05.

### Validation of Dazu black goats by quantitative real-time polymerase chain reaction

We focused on differentially expressed genes with high fold of difference, absolute log2 (FC) greater than 1, high significance (p<0.05), and absolute abundance (FPKM ≥50). On the basis of satisfying these conditions, we randomly selected 12 DEGs in the hypothalamus group, 15 in the pituitary group and 15 in the liver group for quantitative real-time PCR (qRT-PCR) validation to confirm the results of transcriptome sequencing. The primers specific for the DEGs were designed using Primer Premier 5 software, and the specificity of the primers was verified online at the NCBI website (https://www.ncbi.nlm.nih.gov/). Primer sequences and related information are shown in Supplementary Table S1. A PrimeScript RT Reagent Kit (TaKaRa, Tokyo, Japan) was used to synthesise cDNA from 1 μg of total RNA for each sample. Next, qPCR was performed using the TB GreenTM Premix Ex TaqTM II cDNA Synthesis Kit on a real-time PCR instrument (Bio-Rad CFX96, USA). The relative expression level of each gene was normalised to the endogenous control gene GAPDH, and expression ratios were calculated using the 2^−ΔΔCT^ method.

### Statistical methods

Differences in average daily weight gain; anti-SS antibody titres; concentrations of GHRH, SS, IGF-1, GH, and ghrelin; and expression ratios of DEGs between the immunised and negative control groups were analysed using GraphPad Prism8.0 software. we use Shapiro-Wilknormality test for normal distribution. For data that conforms to normal distribution we perform the unpaired t test, based on the F test in the result we determine whether the data conforms to variance homogeneity. Data with inhomogeneous variance were analyzed using the unpaired t test with Welch’s correction. those that did not conform to a normal distribution were analyzed using the Man-Whitney test. Data are expressed as the means±SD, p<0.05 was considered statistically significant and p<0.01 was considered a highly significant difference.

## RESULTS

### Antibody titre

Table 2 shows that the SS-specific antibody titres in the serum of the immunised group of goats were significantly higher than those of the negative control group (p<0.05) at the 4th week after initial immunisation. Consistent with this trend, the SS-specific antibody titres in the serum of the immunised group of goats were significantly higher than those of the negative control group (p<0.05) at the 8th week after the initial immunisation. This finding indicates that the SS DNA vaccine is effective in immunising goats.

### Changes in growth-related hormones in the serum of experimental bucks

**Figure 1:**
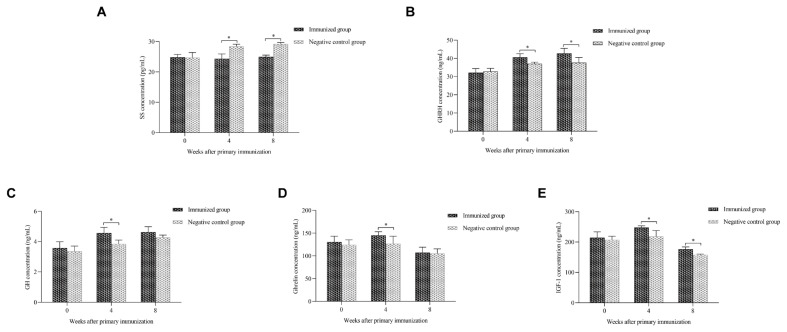
Serum somatostatin (pg/mL), GHRH (ng/mL), GH (ng/mL), Ghrelin (ng/mL), and IGF-1 (ng/mL) concentrations in the immunized and negative control groups at weeks 0, 4, and 8 after initial immunization. Data are expressed as mean±standard deviation; * p<0.05. GHRH, GH-releasing hormone; GH, growth hormone; IGF-1, insulin growth factor 1.

As shown in Figure 1, SS concentrations in serum were significantly lower in the immunised group than in the negative control group at the 4th and 8th weeks after initial immunisation (p<0.05). The concentration of GHRH secreted by the hypothalamus was significantly higher in the immunised group of goats than in the negative control group at the 4th and 8th weeks after the initial immunisation (p<0.05). It’s not the only case, GH concentrations in the serum followed the same trend as GHRH (p<0.05). The concentration of ghrelin secreted by the peripheral in the serum of the immunised group were significantly higher than those of the negative control group at the 4th week after the initial immunisation (p<0.05). However, no significant difference in serum ghrelin concentrations was observed between the immunised and negative control groups (p>0.05) at the 8th week. In addition, IGF-1 concentrations were significantly higher in the immunised group than in the negative control group at the 4th and 8th weeks after initial immunisation (p<0.05).

### Average daily weight gain

The daily weight gain of bucks in the immunised and negative control groups was statistically analysed separately, and the results of the analysis are shown in Table 3. The mean daily weight gain of bucks in the immunised group was significantly higher (p<0.05) than that in the negative control group at the 4th week after the initial immunisation. Similarly, the average daily weight gain of bucks in the immunised group was significantly higher (p<0.05) at the 8th week after the initial immunisation.

### RNA sequencing overview and sequencing data quality assessment

**Figure 2:**
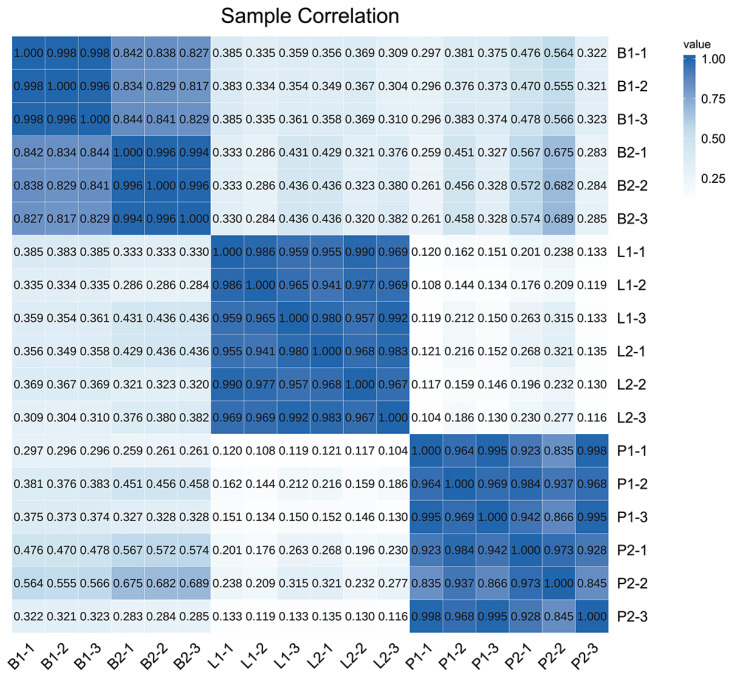
Pearson correlation analysis of B1, B2, L1, L2, P1, and P2 groups. B1-1, B1-2, and B1-3 were hypothalamus samples from 3 goats in the immunised group; P1-1, P1-2 and P1-3 were pituitary gland samples from 3 goats in the immunised group; L1-1, L1-2, and L1-3 were tissue samples from the livers of 3 goats in the immunised group. Negative control group hypothalamic samples named B2-1, B2-2, and B2-3; Pituitary gland samples were P2-1, P2-2, and P2-3; Liver samples were L2-1, L2-2, and L2-3.

In this study, 18 cDNA libraries of hypothalamus, pituitary and liver from different treatment groups (B1-1, B1-2, B1-3, B2-1, B2-2, B2-3, P1-1, P1-2, P1-3, P2-1, P2-2, P2-3, L1-1, L1-2, L1-3, L2-1, L2-2, and L2-3) were constructed and sequenced to generate 802,521,446 raw unique read segments. We used Fastp to filter the raw data prior to subsequent analysis for ensuring the quality of the sequencing data. After all reads containing adapters, reads containing N ratios greater than 10%, all A-base reads and low-quality reads were removed, 800,967,320 clean reads were obtained. The clean reads were then subjected to basic mass analysis, which showed that all 18 samples had Q20 values above 97% and Q30 values above 92%. The results were plotted within the goat reference genome, and at least 86% of the clean reads were aligned to all samples. The distribution of clean reads for all libraries in different regions showed 76.22% to 91.01% annotated exons, 5.15% to 18.33% introns and 3.71% to 6.23% intergenic regions. To verify the biological replication quality of B1, B2, L1, L2, P1, and P2, these correlation coefficients were visualised in the form of heatmaps using the Pearson correlation method (Figure 2). As observed from the heatmap, the correlations for all these samples were high, which ranged from 0.82 to 0.99. The abovementioned results indicate that the sequencing data meet the requirements and can be used for further bioinformatics analysis.

### Regulation of genes related to the hypothalamic–pituitary–liver axis in Dazu black goats by somatostatin DNA immunisation

**Figure 3:**
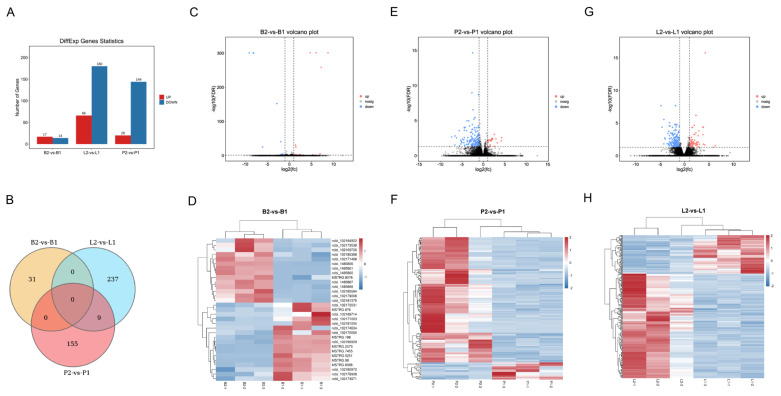
The differential expression analysis of mRNAs. (A) Differential gene statistics of B2_vs_B1, L2_vs_L1 and P2_vs_P1 groups; (B) Venn diagram showing differential expression mRNAs B2_vs_B1, L2_vs_L1 and P2_vs_P1 groups; (C) Volcano map of B2_vs_B1; (D) Hierarchical clustering heatmap of B2_vs_B1; (E) Volcano map of P2_vs_P1; (F) Hierarchical clustering heatmap of P2_vs_P1; (G) Volcano map of L2_vs_L1; (H) Hierarchical clustering heatmap of L2_vs_L1. B2_vs_B1, P2_vs_P1 and L2_vs_L1 groups represent hypothalamus, pituitary and liver tissue samples from goats in the negative control and immunised groups, respectively. B1-1, B1-2, and B1-3 represent the hypothalamus of 3 goats in the immunised group; B2-1, B2-2, and B2-3 represent the hypothalamus of 3 goats in the negative control group; P2-1, P2-2, P2-3 and P1-1, P1-2, P1-3 represent the pituitary glands of 3 goats in the negative control and immunised groups, respectively; L2-1, L2-2, L2-3 and L1-1, L1-2, L1-3 represent the liver tissue of 3 goats in the negative control and immunised groups, respectively.

The analysis of DEGs showed that B2_vs_B1, P2_vs_P1, and L2_vs_L1 produced 31 (17 up and 14 down), 164 (20 up and 144 down) and 246 (66 up and 180 down), respectively, and the results are shown in Figure 3A. The volcano plot visually represents the overall distribution of DEGs in B2_vs_B1, P2_vs_P1, and L2_vs_L1 (Figures 3C, 3E, 3G). The cluster analysis showed that the DEGs of B2_vs_B1, P2_vs_P1, and L2_vs_L1 were well clustered into two classes, which implies that the data were repeatable (Figures 3D, 3F, 3H).

### Functional annotation and classification of the Dazu black goats

**Figure 4:**
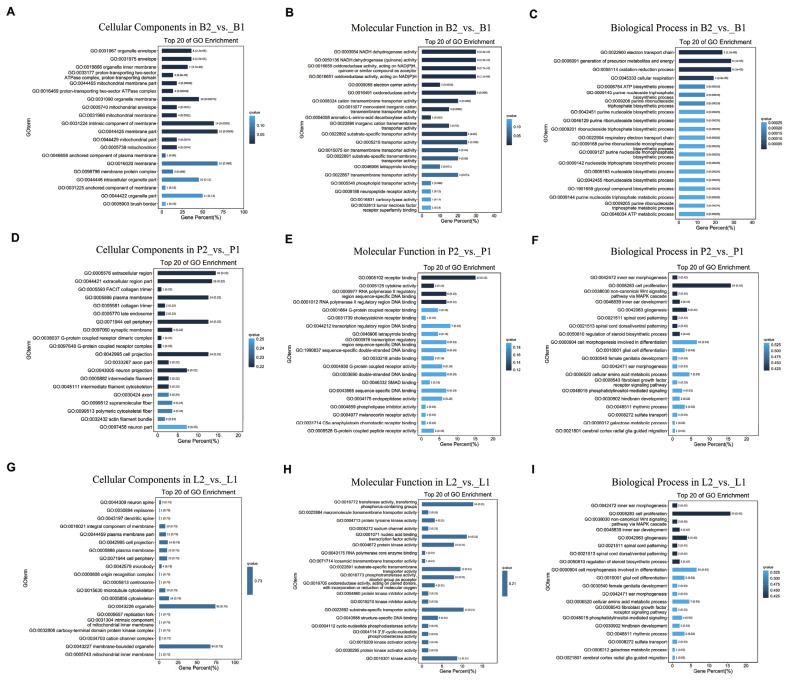
Gene ontology (GO) analysis of differential expression genes. (A) The top 20 significance terms of cellular components in B2_vs_B1; (B) The top 20 significance terms of molecular function in B2_vs_B1; (C) The top 20 significance terms of biological process in B2_vs_B1; (D) The top 20 significance terms of cellular components in P2_vs_P1; (E) The top 20 significance terms of molecular function in P2_vs_P1; (F) The top 20 significance terms of biological process in P2_vs_P1; (G) The top 20 significance terms of cellular components in L2_vs_L1; (H) The top 20 significance terms of molecular function in L2_vs_L1; (I) The top 20 significance terms of biological process in L2_vs_L1. B1 represents the hypothalamus of goats in the immunised group, and B2 represents the hypothalamus of goats in the negative control group; P1 represents the pituitary gland of the immunised group goats, and P2 represents the pituitary gland of the negative control goats; L1 represents the liver of goats in the immunised group, and L2 represents the liver of goats in the negative control group.

GO and KEGG analyses were performed on the B2_vs_B1, P2_vs_P1, and L2_vs_L1 datasets. We performed GO functional enrichment analyses to further understand the functions of DEGs in the hypothalamus, pituitary and liver of bucks. In the B2_vs_B1 dataset, the 31 DEGs were classified into the following three functional categories: biological processes, cellular components and molecular functions (Figure 4A, 4B, 4C). In the category of biological processes, DEGs were assigned to the electron transport chain, generation of precursor metabolites and energy, oxidation-reduction process and cellular respiration. In the category of cellular components, membrane part, intrinsic component of membrane, organelle membrane, organelle envelope, mitochondrial envelope and mitochondrial membrane were the main keywords. Amongst the molecular functional categories, most of the DEGs were associated with NADH dehydrogenase activity, oxidoreductase activity, cation transmembrane transporter activity, substrate-specific transporter activity and aromatic-L-amino-acid decarboxylase activity. All DEGs of P2_vs_P1 were also grouped into the same main functional category (Figures 4D, 4E, 4F). Single-multicellular organism process, single-organism developmental process, multicellular organism development, developmental process and cation transport were the most representative functional words in the biological process category. Receptor binding, cytokine activity, RNA polymerase II regulatory region sequence-specific DNA binding and RNA polymerase II regulatory region DNA binding were the top four functional words in molecular functions. In addition, the functional words classified as cellular components by DEGs were mainly extracellular region, plasma membrane, cell periphery, cell projection and synaptic membrane. In addition, L2_vs_L1 DEGs were highly enriched in organelle, membrane-bound organelle, neuron spine and replisome fractions. Transferase activity, transferring phosphorus-containing groups, nucleic acid binding transcription factor activity and protein kinase activity were highly enriched. In biological processes, DEGs were extremely enriched in cell proliferation, gliogenesis, inner ear morphogenesis and the noncanonical Wnt signalling pathway via the MAPK cascade (Figures 4H, 4I, 4J).

**Figure 5:**
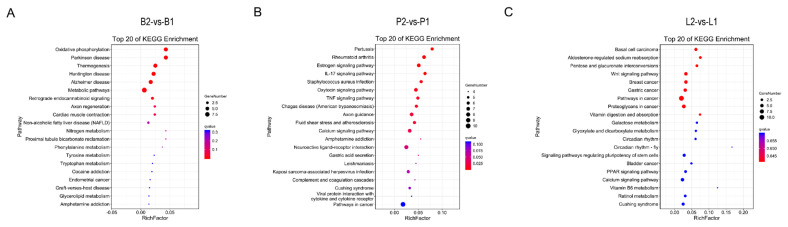
A bubble plot of Kyoto encyclopedia of genes and genomes enriched pathways. (A) B2_vs_B1; (B) P2_vs_P1; (C) L2_vs_L1. B1 represents the hypothalamus of goats in the immunised group, and B2 represents the hypothalamus of goats in the negative control group; P1 represents the pituitary gland of the immunised group goats, and P2 represents the pituitary gland of the negative control goats; L1 represents the liver of goats in the immunised group, and L2 represents the liver of goats in the negative control group.

Pathway significant enrichment analysis was performed using the KEGG database to mine biochemical metabolic pathways and signal transduction pathways associated with DEGs. The results showed that the DEGs in B2_vs_B1 were mainly involved in metabolic pathways, oxidative phosphorylation, thermogenesis and Parkinson’s disease. The DEGs in P2_vs_P1 were mainly involved in the calcium signalling pathway, fluid shear stress and atherosclerosis, axon guidance, the oxytocin signalling pathway and the estrogen signalling pathway. The DEGs in L2_vs_L1 were mainly involved in aldosterone-regulated sodium reabsorption, pentose and glucuronate interconversions, the Wnt signalling pathway, vitamin digestion and absorption, galactose metabolism, glyoxylate and dicarboxylate metabolism and retinol metabolism (Figures 5A–5C).

### Validation of transcriptomic sequencing results by quantitative real-time polymerase chain reaction

**Figure 6:**
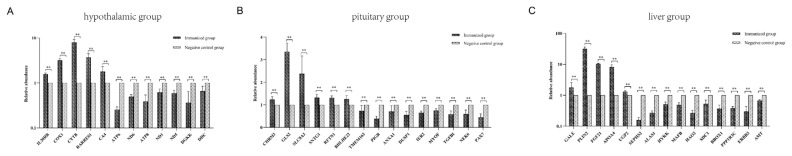
Verification of the selected differentially expressed genes (DEGs) identified in B2_vs_B1 group (A), P2_vs_P1 group (B), and L2_vs_L1 group (C) by quantitative real-time polymerase chain reaction. B1 represents the hypothalamus of goats in the immunised group, and B2 represents the hypothalamus of goats in the negative control group; P1 represents the pituitary gland of the immunised group goats, and P2 represents the pituitary gland of the negative control goats; L1 represents the liver of goats in the immunised group, and L2 represents the liver of goats in the negative control group. * p<0.05; ** p<0.01.

A total of 246, 164, and 12 of the 31 hypothalamic group DEGs (*IL20RB*, *COX1*, *CYTB*, *RARRES1*, *CA4*, *ATP6*, *ND6*, *ATP8*, *ND1*, *ND5*, *DGKK*, and *DDC*) were randomly selected for verification by qRT-PCR to confirm the reliability of the transcriptomic sequencing data. Fifteen of the 164 pituitary group DEGs (*CHRM3*, *GLS2*, *SLC8A3*, *SNTG1*, *RFTN1*, *BHLHE22*, *TMEM163*, *PIGR*, *ANXA1*, *DUSP1*, *IER2*, *MYOF*, *TGFBI*, *NEK6*, and *PAX7*) were randomly selected for verification by qRT-PCR. Fifteen of the 246 liver group DEGs (*GALE*, *PLIN2*, *FGF21*, *APOA4*, *UGP2*, *SEPHS2*, *ALAS1*, *HYKK*, *MAFB*, *HAO2*, *SDC1*, *BBOX1*, *PPP1R3C*, *ERBB3*, and *AMT*) were randomly selected for verification by qRT-PCR. Compared with transcriptome sequencing analysis, similar expression patterns of these representative genes were observed by qRT-PCR (Figure 6).

## DISCUSSION

Ensuring successful immunisation of experimental bucks with recombinant oral SS-14 DNA vaccine was a prerequisite for this study. ELISA results showed that anti-SS antibody titres in the immunised group were significantly higher than those in the negative control group, and immunisation with the SS-14 vaccine successfully induced a humoral immune response (Table 2; Figure 1). The studies presented here suggested that SS DNA immunisation affected the secretion of hormones associated with the hypothalamic–pituitary–liver axis. GH is secreted by the pituitary gland and is a key hormone in the regulation of animal growth [17]. It promotes protein synthesis and the processes involved in regulating lipid and glucose metabolism, which improves the growth of muscle, bone and other tissues [18]. SS can inhibit GH synthesis and secretion, whilst GHRH in the hypothalamus promotes pituitary GH release [19]. In addition, GH is subject to feedback inhibition by peripheral tissue-derived IGF-1. SS/GHRH-GH-IGF-1 forms a hormonal regulatory axis that affects differentiation, proliferation, metabolism and other functions in nearly all tissues [20]. Many studies have found that ghrelin, which is a hormone secreted by the stomach, also has very powerful functions [21]. Current research has focused on ghrelin’s ability to stimulate appetite, stimulate GH release from the anterior pituitary and regulate glucose and energy homeostasis in the animal body [22,23]. In the present study, SS-14 DNA vaccine immunisation resulted in a significant increase in serum concentrations of GHRH, GH, and IGF-1 in goats (Figure 1), which is consistent with that in previous studies [5]. However, ghrelin concentrations in goats’ serum were significantly increased at the 4th week after initial immunisation with the SS-14 DNA vaccine (Figure 1), whilst ghrelin concentrations were insignificantly different between the immunised and negative control groups at the 8th week after oral administration of the SS-14 DNA vaccine. It may be that ghrelin is mainly produced by peripheral organs, and as the time of SS vaccination in goats progresses, feedback signals are transmitted through the hypothalamic-pituitary-adrenocortical axis [24]. Therefore, the SS-14 DNA vaccine increased the level of anti-SS antibodies produced in goats, neutralised endogenous SS, blocked the binding of SS to specific receptors and promoted the production of various hormones, such as GHRH, IGF-1, and GH; thus, it increased the growth rate of DBGs [25].

In the meantime, this study used transcriptome analysis of the effect of SS active immunisation on the expression of genes related to the hypothalamic–pituitary–liver axis in DBGs, which revealed the broad functions of DEGs in various biological processes. RNA-seq analysis of hypothalamus of oral SS-14 DNA vaccine and negative control DBGs identified 31 DEGs, and GO enrichments were observed in the mitochondrial envelope, aromatic-L-amino-acid decarboxylase activity and generation of precursor metabolites and energy. RNA-seq analysis of pituitary of oral SS-14 DNA vaccine and negative control DBGs identified 164 DEGs, and GO enrichments were observed in the extracellular region, receptor binding and multicellular organism development. A total of 246 DEGs were detected in the liver of oral SS-14 DNA vaccine and negative control DBGs by RNA-seq, and GO enrichments included the membrane-bound organelle, transferase activity and cell proliferation. These results suggest that the SS-14 DNA vaccine significantly affects the regulation of nutrient metabolism in the hypothalamic–pituitary–liver axis. In this study, a number of DEGs were selected for qRT-PCR validation in different groups. Compared with transcriptome sequencing analysis, similar expression patterns of these representative genes were observed by qRT-PCR (Figure 6).

RNA-seq analysis of hypothalamus of oral SS-14 DNA vaccine and negative control DBGs identified 31 DEGs, and GO enrichments were observed in the mitochondrial envelope, mitochondrial membrane, organelle envelope and intrinsic component of membrane. These results imply that organelle structures such as mitochondria in the hypothalamus are firstly affected during immunisation of goats with the SS-14 DNA vaccine [26]. The GO term aromatic-L-amino-acid decarboxylase activity and generation of precursor metabolites and energy were also enriched. *ATP6*, *ATP8*, *ND1*, *ND5*, *ND6*, *DDC*, and *DGKK* were downregulated by SS-14 DNA vaccine immunisation treatment, whilst *CYTB*, *COX1*, *IL20RB*, and *RARRES1* were upregulated and further verified by qRT-PCR (Figure 6). In this study, we observed for the first time that SS active immunity affects the expression of genes related to electron transport in the mitochondrial respiratory chain of the hypothalamus [27]. *ATP6*, *ATP8*, *ND1*, *ND5*, *ND6*, and *COX1* are protein-coding genes of respiratory chain complex I [28], which are involved in mitochondrial electron transport, located in the mitochondrial membrane and play a key role in energy production and metabolism [28].

In the meantime, oxidative phosphorylation and metabolic pathways were found to be closely related to growth and development regulation by KEGG pathway analysis (Figure 5A). Although the main function of oxidative phosphorylation is to produce energy and maintain redox homeostasis, an increasing number of studies have confirmed that anterior pituitary GH affects oxidative phosphorylation and is closely related to individual development [29]. *CYTB*, which is located in mitochondria, is an oxidative phosphorylation-related protein that is involved in several processes, including electron transport coupled proton transport and response to glucagon [30]. A study also showed that polymorphisms in *CYTB* genes of three Chinese donkey breeds had an effect on growth traits [31]. Metabolic pathways are central regulators of many cellular processes, including cell metabolism, growth and proliferation, but overactivation during active vaccine immunisation may facilitate their replication. *DDC* was enriched in metabolic pathways, and one study reported that *DDC* synthesises serotonin in the developing mouse heart, encoded by Ddc_exon1a; evidence from the Ddc_exon1a knockout mouse model suggested that it mediated the growth of the developing myocardium, and myocardial thinning was observed in the small number of mutant mice examined [32]. The expression of *DDC* in the present changes that occurred in this study may be related to the growth of DBGs.

DEGs belonging to the GO term developmental process and cell proliferation were observed in the pituitary and liver groups. Nine of the same DEGs were present in the pituitary and liver groups, including *DUSP1*, *PRSS53*, *NANOS1*, and *TPX2* (Figure 3B). The protein encoded by *DUSP1* is a phosphatase with dual specificity for tyrosine and threonine, and *DUSP1* can dephosphorylate the MAP kinase MAPK1/ERK2, which results in its involvement in several cellular processes [33]. *PRSS53* has been demonstrated to enhance serine-type endopeptidase activity, which is involved in protein hydrolysis [34]. The expression of *DUSP1* and *PRSS53* can be reduced in the pituitary by treatment with the SS-14 DNA vaccine, which in turn regulates amino acids that are important in nutrient metabolism processes. We also found that *NANOS1* and *TPX2*, which are associated with male reproduction, were downregulated in the pituitary and liver groups by treatment with the SS-14 DNA vaccine. *NANOS1* encodes a CCHC-type zinc finger protein that is involved in regulating translation as a posttranscriptional repressor, and mutations in *NANOS1* are associated with a lack of germ cells in the testis or severe oligozoospermia [35]. These results suggest that treatment of bucks with the SS-14 DNA vaccine may affect the function of germ cells in the testis and changes in protein kinase activity, which may impact the reproductive performance of bucks.

KEGG pathway analysis showed that DEGs in the pitu itary and liver groups were enriched in growth- and nutrient metabolism-related pathways such as the calcium signalling pathway, retinol metabolism and vitamin digestion and absorption (Figures 5B, 5C). In the present study, *CHRM3* was found to be upregulated in the pituitary gland after treatment with the SS-14 DNA vaccine in bucks. The expression of *LART* was downregulated. *CHRM3* belongs to the calcium signalling pathway, which is present in the genomic copy number variable region of yak populations and overlaps with quantitative trait loci related to meat quality and growth [36]. *LRAT* is involved in retinol metabolism and vitamin digestion and absorption. Reduced retinyl ester levels in the ovaries of mice treated with follicle-stimulating hormone and inhibition of *LRAT* expression catalyse the esterification of retinol [37]. This reaction is an important step in vitamin A metabolism in the visual system. Functional hepatic stellate cells co-expressing *LRAT* and *CRBP-1* also continue to maintain the ability to store vitamin A [38].

There were several major limitations in this study. One weakness was the small sample size and single exposure dose. The use of more multi-dose samples in different treatment groups would improve statistical power and facilitate the identification of more DEGs, thereby elucidating underlying molecular mechanisms. Not exploring the function of the identified key differential genes associated with growth was also a shortcoming. Since the subsequent experiments in this study were based on the improvement of goat production performance, it is necessary to investigate the functions of key differentially expressed genes, such as *CYTB*, *DUSP1*, and *CHRM3*, and to analyse the molecular mechanisms by which the hypothalamus-pituitary-liver axis affects the production performance of goats in the future studies.

## CONCLUSION

In this study, we explored the effect of somatostatin active immunisation on gene expression in the hypothalamus-pituitary-liver axis of Dazu black goats using the transcriptome. Dazu black goats were annotated for metabolic pathways, calcium signalling pathways, vitamin digestion and absorption. In conclusion, somatostatin DNA vaccine active immunisation increased the growth rate of Dazu black goats by altering growth-related hormone concentrations and modulating the expression of growth-related genes in the hypothalamic–pituitary–liver axis. Our results provide a reference for exploring the molecular mechanisms by which somatostatin promotes Dazu black goats growth performance.

## Figures and Tables

**Table 1:** Nutrient levels (dry matter basis) for commercial full-price pellets

Nutrient level	Immunised group	Negative control group
Digestible energy (DE, MJ/kg)	12.14	12.14
Crude protein (CP, %)	16.63	16.63
Crude fat (CF, %)	2.04	2.04
Neutral detergent fiber (NDF, %)	37.91	37.91
Acid detergent fiber (ADF, %)	24.80	24.80
Crude ash (Ash, %)	10.31	10.31
Calcium (Ca, %)	1.08	1.08
Phosphorus (P, %)	0.64	0.64

CP, CF, NDF, ADF, Ash, Ca, P were analyzed values, and DE is a calculated value.

**Table 2:** Anti-somatostatin antibody titers of Dazu black goats in the immunised and negative control groups at weeks 4, and 8 after initial immunisation

Weeks after initial immunisation(w)	Antibody titers	

	Immunised group	Negative control group
4w	500.00±173.21^a^	0.00±0.00^b^
8w	1,000.00±346.41^a^	0.00±0.00^b^

Data are presented as means±standard deviation.

a,b Within a row, means with different superscripts differed (p<0.05).

**Table 3:** Changes in daily weight gain of Dazu black goats after immunisation with somatostatin DNA vaccine

Weeks after Initial immunisation (w)	Average daily gain	

	Immunised group	Negative control group
4w	119.73±15.59^a^	86.34±10.32^b^
8w	114.45±6.70^a^	84.51±5.79^b^

Data are presented as means±standard deviation.

a,b Within a row, means with different superscripts differed (p<0.05).

## Data Availability

The following information was supplied regarding data availability: The PCR data are available in the Supplemental Files. The transcriptome sequencing data supporting the results of this study will be available at National Center for Biotechnology Information at https://www.ncbi.nlm.nih.gov/sra/PRJNA999054 after a 5-year embargo period from the date of publication.

## SUPPLEMENTARY MATERIAL

**Supplementary Table S1**. Primers used for qRT-PCR validation of differentially expressed genes
