# Supplementary material for: Immunisation of the somatostatin gene alters hypothalamic-pituitary-liver gene expressions and enhances growth in Dazu black goats
Source: Anim Biosci. 2024 Jun 26;37(11):1987–99. doi: 10.5713/ab.24.0121 (PMC11541029; doi:10.5713/ab.24.0121)
Supplement: Supplementary file 1 [file ab-24-0121-Supplementary-Table-1.pdf]

Supplementary Table 1. Primers used for qRT-PCR validation of differentially expressed genes

Gene name	Forward primer (5′-3′)	Reverse primer (5′-3′)
IL20RB	ACGGAGGACATCACTGCTACTG	AGGATGGTTGAGTTCTGGTTAAAGC
DGKK	AGCACGGCAACCACAGAATATG	GTTATCATCACCTCACGGCACTG
CA4	CAGTGGGTCGTGGGAAACAATG	GTGCTCAGTCCTCCTCCAGTG
ND1	ACCATACGGCTTACTTCAACCAATC	TGAGGATGTGGCAGGTCGTAAG
ND5	CATCACAATACTCATCCTCGTCACC	CGTCTGTTCGTCCATATCACCATC
ATP6	GCCCACACCACTAATCCCAATG	AGTAAGTGTCCTGCTGTGATGTTG
ATP8	TCAAAGTACGACTTCTACCACAACC	TTTTGTTTCTCAAGGGGTGTTATGC
COX1	TCCAAACGAGAGGTCCTAACTGTAG	TGGGTTCTTCAAATGTGTGGTATGG
RARRES1	CCTGCCGTATTCACTTGGTCTG	CTTCTGGTGTCTGTCGCTCTTG
CYTB	ACTCGGAGACCCAGACAACTATATC	GAATTGATCGTAGGATTGCGTATGC
DDC	TCCCTACTTCTTCGCCTACTTCC	CAGTCCATCATCACCGTCTCAAG
ND6	GGCGGGATGTGGTATTGTGTTG	AGTAGCCATAGCCGTCGTATAACC
SEPHS2	TCAGCGTCAGCCAGAGTATGC	CAACTCCACCGATGATAATCCAAGG
UGP2	CTCAGGATGGTGCTTCTCAGTTC	GTGATGGTGCTGTGGTGAGTATC
PLIN2	GATGCTGTGACGACGACTGTG	TGCCACTGACCACGGACTTG
BBOX1	TGGCAGGATGGTACAGACTCTATC	TCAAGGTAGCAGTCGGGACAC
SDC1	GACCCGCCACCTCCCATC	CCTCCTCAGCCGCCTTCTC
HAO2	CGTCCAGGGCATCATTGTTTCC	CACCACTTCTGTCAAGGCATCG
ALAS1	GATGCCGCACTCTTGTTCTCC	GCACTCCGCTGTTCCGAATC
HYKK	CAGCCTCTGTGTGTCGTACTAAAG	GTAAGTCAGCAGCCTCACCAAG
MAFB	ACCATCACCACCACCACCAAG	CTCCACGCTGCCGCTACC
PPP1RSC	AGGCTCTGCTTGGCTCATTCC	CGTCCTGTGCTCTGGCTTCC
ERBB3	CTATGAGAAGTGTGAGGTGGTGATG	AGGAGAGGTCAGCGTTGTGTC
GALE	GCAATGACTATGACACAGAGGATGG	GCCCGTGCCCAGGTTGTAG
AMT	TGATGGAGAGTCTGGTGGTTGG	CTCGGAAGCGTTGGTCACAATC
FGF21	CTGTGCTGGCTGTCCTCCTG	CTCTGTCTCCTGGGCATCATCC
CHRM3	ACGATGCTGCCGCCTCTC	TGATGAAGGTAGTTTGGTGGAGTTG
GLS2	ACCAGACGCAGCATCAGGATC	CAGCAATAGTGTAGAAGAGCAGGTC
SLC8A3	AAGGTAATTGATGGTGAGGCGTATG	GGCTTCTTCTTCTTCCACAGTCAG
TMEM163	GACCAGCAGAGCACTCATAACAG	GCCAATCAGCACGCCGATG
PIGR	AAAGAACCCAAGAAGGCAAAGAGG	CTGGAGGAGGAAGGTGGTGAAG
ANXA1	TTGCTTTCTCTTGCTAAGGGTGAC	CCTGGTGGTAAGAATGGTAGTGAAC
DUSP1	GACAACCACAAGGCGGACATC	TGAGGTAAGCGAGGCAGATGG
IER2	AGCAGCAGCCTTAGCGATGG	CCCTTCCTCTTCCTCCTCTTCTTC
MYOF	ATGGAGGTGATGAGGACAGGTTG	TGCGTCGGCTGCTCTTCG
PAX7	CCGAGACCGCCTGCTGAAG	CGCCGTCCTCCTCCTTCTTG
TGFBI	TGGTCAGCGGAGGCATTGG	AGCAACAGGTTCCTTATTGACACTC
NEK6	CCTCCTGAAGCAACTGAACCATC	CGCCAGCTCCAGCACAATG
SNTG1	GGGCTGCGGTGGGAGAAG	ACGGCGGTGACTTGGAAGG
RFTN1	GAGCACATGAGCGACCACTTC	GGACCAAGCCAAGCCAGGAG
BHLHE22	CTGCTCCAACGCCCTCCTC	TCTTGCTGCCGCCACTACC

APOA4	CCTATGGCGAGACCTTCAACAAAG	GCCTGGCTCTCCTTCTCCTTG
GAPDH	TTATGACCACTGTCCACGCC	TCAGATCCACAACGGACACG
